# Evaluation of fluopyram for the control of *Ditylenchus dipsaci* in sugar beet

**DOI:** 10.21307/jofnem-2020-071

**Published:** 2020-07-28

**Authors:** Alan Storelli, Andreas Keiser, Reinhard Eder, Samuel Jenni, Sebastian Kiewnick

**Affiliations:** 1 School of Agricultural, Forest and Food Sciences (HAFL), Bern University of Applied Sciences (BFH), Zollikofen, Switzerland; 2 National Competence Centre for Nematology, Agroscope, Wädenswil, Switzerland; 3 Swiss Sugar Beet National Competence Centre (SFZ/CBS), Aarberg, Switzerland; 4 Julius Kuehn Institute, Federal Research Center for Cultivated Plants, Institute for Plant Protection in Field Crops and Grassland, Messeweg 11/12, 38104 Braunschweig, Germany

**Keywords:** *Ditylenchus dipsaci*, Fluopyram, Management, Nematicide, Nematode control, Plant-parasitic nematode, Sugar beet

## Abstract

Fluopyram, a succinate dehydrogenase inhibitor fungicide, has shown potential in controlling *Meloidogyne incognita* and *Rotylenchus reniformis* in tomato. The effectiveness of this compound for the control of *Ditylenchus dipsaci* in sugar beet was evaluated. In this study, laboratory, growth chamber, glasshouse, and field experiments were conducted. In a motility bioassay, the EC_50_ value was determined with 3.00 μg/ml a.i. after 72 h exposure to fluopyram. The growth chamber experiment did not show any effects on *D. dipsaci* penetration rate; however, field experiments revealed a positive effect of fluopyram applied at planting in reducing *D. dipsaci* infectivity. The glasshouse experiment confirmed a limited effect of fluopyram on *D. dipsaci* population development. Under field conditions, despite a reduction of *D. dipsaci* penetration rates in spring, fluopyram was not effective in reducing the population development until harvest. Consequently, *D. dipsaci* densities in plant tissue and soil were high at harvest and not different among treatments. However, root-rot symptoms were significantly reduced at harvest. Fluopyram applied at planting showed good potential to reduce root-rot symptoms caused by *D. dipsaci* in sugar beet. However, for the long-term reduction of nematode populations in soil, further integrated control measures are needed to reduce the risks of substantial yield losses by *D. dipsaci*.

*Ditylenchus dipsaci* (Kuhn) Filipjev, a migratory endoparasite with a worldwide distribution, is ranked no. 5 of the top 10 plant-parasitic nematodes worldwide (Jones et al., 2013). Although primarily infecting onion and garlic, *D. dipsaci* has a wide range of host plants, including sugar beet. All stages of *D. dipsaci* can infect sugar beet through stomata or wounds, followed by feeding on cell contents. Surrounding cells then start to divide and enlarge, resulting in malformation of tissue and consequently leading to severe crop damage. Even at low initial population densities in soil, rapid reproduction with up to six generations per season at 15 to 20°C can lead to severe losses in sugar beet (Yuksel, 1960; Duncan and Moens, 2013). As currently no resistant varieties are available for the control of *D. dipsaci*, chemical control has been the method of choice, but fumigation or use of nematicides are either no longer available or uneconomical worldwide (Jones et al., 2013). Due to the ability of the *D. dipsaci* fourth-stage juveniles (J4) to survive for many years in soil or plant debris (Fielding, 1951), management of this nematode is challenging. Since 2016, the Swiss Federal Office for Agriculture has been implementing an exemption on the use of fluopyram on *D. dipsaci* infected sugar beet fields.

Fluopyram is a succinate dehydrogenase inhibitor fungicide inhibiting fungal respiration (Avenot et al., 2012). The fungicide activity spectrum of fluopyram includes a number of pathogens belonging to Ascomycota (Veloukas and Karaoglanidis, 2012). The acute LD_50_ of fluopyram to rats via oral administration is greater than 2,000 mg/kg (European Food Safety Authority, 2013), as opposed to aldicarb, which is 0.5 to 1.5 mg/kg (Oka et al., 2012). The active ingredient has also nematicidal or nemastatic properties (Faske and Hurd, 2015). Nematicides with fluopyram as the active ingredient are registered in several countries to control plant-parasitic nematodes such as *Meloidogyne incognita* or *Rotylenchus reniformis*. In the USA, fluopyram has been used since 2015 to control nematodes in cotton and peanut. In 2014, fluopyram was registered as a seed treatment to control the soybean cyst nematode, *Heterodera glycines* (Beeman and Tylka, 2018). Currently available studies also demonstrated how fluopyram affects the development of the nematodes species, *M. incognita*, *M. javanica*, *H. glycines*, *H. schachtii*, and *Pratylenchis loosi* (Hurd et al., 2015; Kim et al., 2016; Beeman and Tylka, 2018; Mohotti et al., 2018; Dahlin et al., 2019; Hajihassani et al., 2019; Ji et al., 2019; Oka and Saroya, 2019; Schleker et al., 2019; Silva et al., 2019).

This study aimed to (i) evaluate the sensitivity of *D. dipsaci* to fluopyram (ii), determine the effect of different concentrations of fluopyram on the penetration rate of *D. dipsaci* into sugar beet seedlings, (iii) determine the effect of different concentrations of fluopyram on the reproduction rates of *D. dipsaci* in sugar beet, and (iv) determine the optimum application strategy for fluopyram under field conditions.

## Materials and methods

### Nematode inoculum and chemical

The *Ditylenchus dipsaci* population used in all experiments was extracted using Oostenbrink dishes (European and Mediterranean Plant Protection Organization, 2013) from infested sugar beets collected in the Seeland region in Switzerland. Using a fine needle, 2,000 fourth-stage juveniles (J4) and adult nematode stages were hand-picked and transferred to sterile centrifuge tubes. After suspending the nematodes in an antibiotic solution containing 0.1% streptomycin sulfate (w/v) and 0.1% amphotericin-B (w/v), nematodes were inoculated on carrot cylinders (2.5 × 5 cm) and incubated at 20°C in the dark for 50 days (Kühnhold et al., 2006). After extraction from the carrot cylinders, nematodes were stored at 6 to 8°C in the dark up to 20 days until further use.

The nematode population for the inoculation of in vitro, growth chamber, and glasshouse experiments consisted of J2, J3, J4 juveniles, and adults, with a predominance of adults and J4 juveniles.

A suspension concentrate formulation, containing fluopyram at 500 g a.i./L (MOON PRIVILEGE), was supplied by Bayer CropSciences Schweiz AG (Zollikofen, Switzerland). This formulation was already registered for use on tree crops, floriculture crops, and vegetable crops in Switzerland (FOAG, 2019).

### Effect of fluopyram on *D. dipsaci* motility

To evaluate the direct effect of fluopyram on *Ditylenchus dipsaci*, nematodes were incubated for 96 h at 22°C in water containing of 50, 30, 10, 5, 3, 2, 1, and 0 µg/ml of fluopyram. The experiment was conducted in six-well polystyrene plates with each well receiving 2.5 ml of a 2 × concentrated test solution and 2.5 ml of distilled water containing 1,000 *D. dipsaci* (Dutta et al., 2012). Each treatment was replicated three times, and the experiment was conducted four times. After exposure for 24, 48, 72, and 96 h, motile and immotile nematodes were counted using an optical microscope at 100 × magnification. Motile *D. dipsaci* were characterized by their serpentine shape, while immotile nematodes were straight with no visible movement. Additionally, after 72 h exposure to fluopyram, nematodes were washed with water over a 20 µm sieve, and after further 24 h incubation in water, the number of motile/immotile *D. dipsaci* was recorded to determine their recovery in motility (Faske and Hurd, 2015).

### Effect of fluopyram under controlled conditions

The impact of fluopyram on the penetration rate of *D. dipsaci* into sugar beet was investigated in a growth chamber. The treatments were two application methods, either at planting or post-planting (cotyledons horizontally unfolded, BBCH 10), and five concentrations (10, 5, 1, 0.5, and 0.0 µg/ml). The susceptible cultivar Dorena (KWS Saat SE, Germany) was sown into 3-cm-diameter plastic pots filled with 80 ml of soil. The soil was a loam that was steamed, passed through a 2 mm sieve, and mixed with sand (1/1,v/v). Each plastic pot contained one sugar beet seed sown 2 cm deep. Fluopyram was applied to the soil surface of the pots receiving the at-planting application. At 7 days post-planting (dpp), 1 ml fluopyram solution was applied 3 h before nematode inoculation to the sugar beet leaves and soil surface of the pots receiving the post-plant application. Because BBCH 11 (first pair of leaves visible, not yet unfolded) seedlings allow optimal staining of the nematodes in the plant, fluopyram was applied at BBCH 10 instead of BBCH 12 (two leaves unfolded), as done in the glasshouse and field experiments. At BBCH 10, approximately 200 nematodes per seedling were inoculated in 10 µl of 1% carboxymethylcellulose (w/v), which increases the adhesion of nematodes to the leaf-axils. The inoculation droplet was placed between the first pair of true leaves of the seedlings (Kühnhold et al., 2006). At 7 days post-inoculation (dpi), the seedlings were removed from the pots, gently washed, transferred to a 100-ml plastic beaker containing a 0.1% acid fuchsin/lactic acid solution and boiled twice in a microwave oven for 1 min. Stained seedlings were then rinsed to remove the staining solution. The total number of nematodes per seedling was counted using a stereomicroscope at 10 × magnification after maceration in 30 ml tap water using an Ultra Turrax blender (T25 basic, IKA Labortechnik, Germany). The experimental design was a factorial design arranged in randomized complete blocks, each treatment was replicated ten times, and the experiment was conducted twice. The experiment was conducted at 15°C with a 16-h photoperiod and covered with a transparent plastic foil to ensure 95% relative humidity.

The impact of fluopyram on the reproduction rate of *D. dipsaci* in sugar beet was investigated in a glasshouse. The treatments were two application methods, either at planting or post-planting (two leaves unfolded, BBCH 12), and four concentrations (5.0, 1.0, 0.5, and 0.0 µg/ml). The susceptible cultivar Dorena (KWS Saat SE, Germany) was sown in 1 L plastic pots filled with 800 ml soil. The soil was a loam that was steamed, passed through a 2 mm sieve, and mixed with sand (1/3, v/v). The aim to obtain well-developed roots motivated the higher rate of sand in the soil mixture compared to the experiment conducted in the growth chamber. Each plastic pot contained one sugar beet seed sown 2 cm deep. Fluopyram was applied to the soil surface of the pots receiving the at-planting application. At BBCH 10, approximately 200 nematodes per seedling were inoculated in 10 µl of 1% carboxymethylcellulose (w/v). The inoculation droplet was placed between the first pair of true leaves of the seedlings (Kühnhold et al., 2006). At BBCH 12, 1 ml fluopyram solution was applied to the sugar beet leaves and soil surface of the pots receiving the post-plant application. The experiment was started in a growth chamber at 15°C with a 16-h photoperiod and covered with a transparent plastic foil to ensure 95% relative humidity to ensure nematode infection. At 7 dpi, the plants were transferred for higher temperatures to a glasshouse at 20 ± 1°C with an 18-h photoperiod. At 50 dpi, the plants were removed from pots, washed, weighed, and cut in 0.5 cm pieces. Nematodes were extracted from the sliced plant material using Oostenbrinck dishes (European and Mediterranean Plant Protection Organization, 2013). The number of *D. dipsaci* per sugar beet plant was determined by counting 3 × 1 ml aliquots from 200 ml total volume using a stereomicroscope. The experimental design was a factorial design arranged in randomized complete blocks, each treatment was replicated 10 times, and the experiment was conducted twice.

### Field experiments

The field experiment, conducted in 2016, was set up as randomized complete block designs with four treatments: fluopyram applied in-furrow at planting at 250 g a.i./ha, in-band at BBCH 12 at either 125 or 250 g a.i./ha, and an untreated control. According to the Swiss standard, fluopyram was applied with 300 L/ha. The timing of the post-plant application (BBCH 12) was 28 dpp. The two widely used cultivars Samuela (KWS Saat SE, Germany) and Hannibal (Strube GmbH, Germany) were planted at two locations in Kappelen and Bargen, respectively. Both cultivars are similar in their susceptibility to *D. dipsaci* compared to the cultivar Dorena. Sugar-beet plant samples (10 plants/plot and sampling date) were randomly collected 27 and 62 dpp. Plants collected at 27 dpp were analyzed by staining with 0.1% acid fuchsin (see above), whereas the Oostenbrink dish technique was used to determine nematode numbers in plants collected 62 dpp.

Population densities of *D. dipsaci* (*Pf/Pi*) in these field sites were investigated by taking random soil samples seven days before planting and at harvest. In all, 10 subsamples/plot of harvested sugar beets were randomly collected, and the number of *D. dipsaci* in 100 g sugar beet hypocotyl tissue was determined by extraction using Oostenbrink dishes.

The effect of fluopyram on root-rot symptom development on sugar beets was determined by de-heading 20 beets/plot down to the crown surface and slicing them vertically until the bottom of the root. The percentage of root volume showing rot symptoms was determined using an index with 0 = no rot visible, 1 = ≤ 10% of rotten root, 2 = 11 to 33% of rotten root, 3 = 34 to 66% of rotten root, and 4 = 67 to 100% of rotten root.

In 2017, field experiments were repeated, but warm and dry conditions in spring were unfavorable to *D. dipsaci* development. Consequently, nematode populations were low and did not lead to any damage symptoms. Therefore, we did not use the data for the year 2017.

### Statistical analysis

Data for nematode motility, infectivity (*D. dipsaci*/seedling), reproduction under glasshouse conditions (*D. dipsaci*/plant 50 dpi), soil populations (*Pf*/(*Pi* + 1)), reproduction in the field (*D. dipsaci*/100 g beet crown tissue), and root rot symptoms were tested for normality (Shapiro-Wilk normality test) and homogeneity of variances (Bartlette and Levene Test). Data obtained in the growth chamber and in the glasshouse were subjected to a two-way analysis of variance. The data from repeated experiments were pooled after confirming the absence of variation between the experiment replications. Data from field experiments were analyzed individually for each site and subjected to a Friedman test. Tukey multiple comparisons of means were performed as a post hoc test (Lenth, 2016). Statistical analysis, as well as graphs, were performed with R.

A logistic dose-response curve was used in the motility bioassay to describe the relationship between inactive *D. dipsaci* after 24, 48, 72, 96 h, and 72+rinse (*y*) and the concentration (*x*) of the active ingredient fluopyram (Hutchinson et al., 1999; Kiewnick and Sikora, 2006). The logistic curve can be expressed as follows:

(1) $$y=c+{\left[\frac{d-c}{1}+\left(\frac{x}{{\mathrm{EC}}_{50}}\right)\right]}^{b},$$

(1)where *c* and *d* are the lower and upper limits of the curve, respectively. The EC_50_ is the concentration reducing the response (*y*) 50% between *d* and *c*, i.e., the point of inflection. The parameter *b* is the proportional slope of the curve around the point of inflection (Ritz and Streibig, 2005). The four-parameter logistic model (Equation (1)) was reduced to a three-parameter model:

(2)y=d/[1+(xEC20)b]

(2)where *d* is the upper limit of response (upper asymptote) (Ritz and Streibig, 2005; Kiewnick and Sikora, 2006). Curve fitting was performed by non-linear regression using the extension package *drc* in R (Ritz et al., 2015).

## Results

### Effect of fluopyram on *D. dipsaci* motility

**Figure 1:**
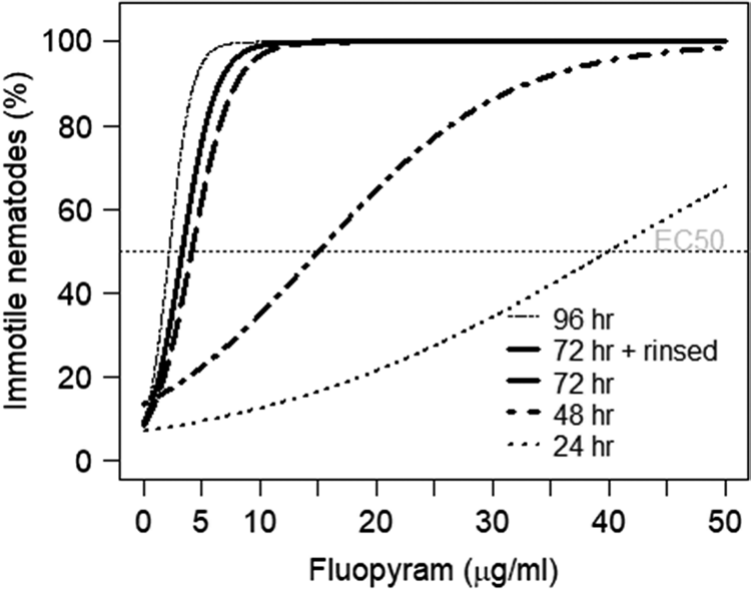
Dose-response curves for immotile *Ditylenchus dipsaci* (%) after 24, 48, 72, and 96 h exposure to concentrations of 0, 1, 2, 3, 5, 10, 30, and 50 µg a.i./ml of fluopyram. Recovery of motility was determined by rinsing the a.i. of nematodes from the 72 h treatment and after further incubation for 24 h in water (= 72 hr + rinsed).

When exposed to fluopyram, the percentage of immotile *D. dipsaci* increased with exposure time and concentration (Fig. 1). After 24 h, only 58% of *D. dipsaci* were immotile at the maximum concentration of 50.0 µg/ml fluopyram, resulting in an EC_50_ value of 46.73 µg/ml (Table 1). After exposure for 48, 72, and 96 h to fluopyram, EC_50_ values were 9.32, 3.98, and 2.02 µg/ml, respectively. After 72 h, only concentrations of 30 and 50 µg/ml fluopyram resulted in 100% immotile *D. dipsaci*. However, after washing *D. dipsaci* free of fluopyram and further incubation in water for 24 h, no increase in motility was observed. The EC_50_ value for the 72 h + rinse was 3.00 µg/ml (Table 1). The percentage of immotile *D. dipsaci* continued to grow with the exposure time for each fluopyram concentration, except for 0 µg/ml. After a further 24 h incubation in water (72 h + rinse), the percentage of immotile *D. dipsaci* continued to grow compared to 72 h exposure to fluopyram. At 3.00 µg/ml a.i., the percentage of immotile nematodes was 40, 76, and 58% after exposure for 72 h, 96 h, and 72 h + rinse, respectively.

**Table 1.** Estimated parameters for non-linear regression of the parameter inactive *Ditylenchus dipsaci* at different fluopyram exposure times (y) and concentrations (± SE).

Parameter				
Exposure time (*y*)	*b*	EC_50_ (µg/ml)^a^	*c*	*d*
24 h	−1.17 ± 0.65	46.73 ± 88.19	3.54 ± 3.19	108.50 ± 119.46
48 h	−1.68 ± 0.68	9.32 ± 2.27	5.11 ± 4.70	96.03 ± 12.80
72 h	−2.43 ± 0.48	3.98 ± 0.33	7.62 ± 3.52	101.52 ± 4.01
96 h	−2.27 ± 0.34	2.02 ± 0.16	5.56 ± 3.73	101.68 ± 3.23
72 h + rinsed	−2.11 ± 0.27	3.00 ± 0.22	4.98 ± 2.96	101.96 ± 3.53

**Notes:** ^a^EC_50_ is 50% effective concentration in µg fluopyram/ml water, where *c* and *d* are the lower and upper limits of the curve, and *b* is the proportional slope of the curve around the point of inflection.

### Effect of fluopyram under controlled conditions

**Figure 2:**
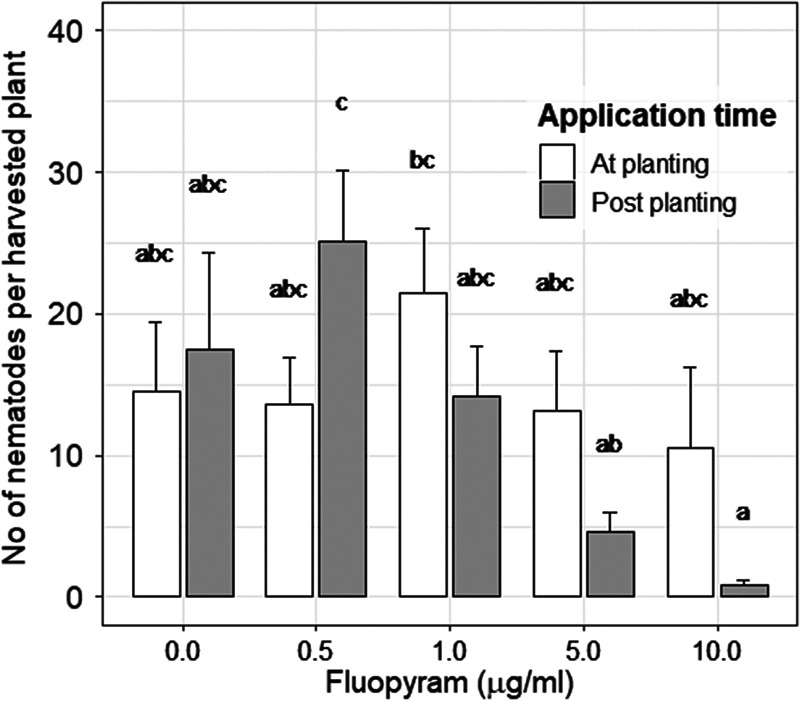
Effect of different fluopyram concentrations (0.0, 0.5, 1.0, 5.0, 10.0 µg/ml) applied either at planting or at BBCH 10 (cotyledons horizontally unfolded) on the number of *Ditylenchus dipsaci* per seedling (BBCH 11, first pair of leaves visible - not yet unfolded) seven days post-inoculation. Different letters over bars indicate significant differences at *α* =0 .05, according to the Tukey multiple comparisons of means.

In the growth chamber experiment, there was a significant effect of the fluopyram concentration (*p *< 0.001), but no effect of the application time (*p* = 0.597) and no interaction between fluopyram concentration and application time (*p* = 0.224) on the number of penetrated *D. dipsaci* in sugar beet seedlings at 7 dpi (Fig. 2). When fluopyram was applied to the leaves and soil surface at BBCH 10 with 10.0 µg/ml or 5.0 µg/ml per plant, the number of *D. dipsaci* penetrating sugar beet seedlings was significantly lower compared to a concentration of 0.5 µg/ml (*p* < 0.001). Fluopyram application did not affect the rate of *D. dipsaci* penetration compared to the untreated plants (water control).

In the glasshouse experiment, where the reproduction of *D. dipsaci* in sugar beet plants was determined at 50 dpi, population densities of 12,344 to 27,681 nematodes per plant were found in the untreated control plants (Table 2). Fluopyram treatment did not affect the final number of *D. dipsaci*/plant. No significant effect of the fluopyram concentration and application time were found (*p* > 0.05). Fluopyram at rates of 0.5, 1.0, or 5.0 µg a.i./plant did not reduce *D. dipsaci* densities in plants, neither when applied at planting nor at BBCH 12 (Table 2).

**Table 2.** Effect of fluopyram applied at-planting or at BBCH 12 (two leaves unfolded) at concentrations of 0.0, 0.5, 1.0, and 5.0 µg/ml on the number of *Ditylenchus dipsaci* (± SE) per sugar beet plant 50 days post-planting in the glasshouse.

	Fluopyram application	
Fluopyram concentration (a.i.)	at planting	BBCH 12
0.0 (µg/ml)	27,681 ± 34,208	12,344 ± 18,727
0.5 (µg/ml)	16,782 ± 28,521	13,750 ± 22,242
1.0 (µg/ml)	23,901 ± 37,066	8,596 ± 7,637
5.0 (µg/ml)	20,315 ± 22,765	14,126 ± 22,566

**Note:** No difference among the fluopyram concentrations, according to the Tukey multiple comparisons of means at *α* = 0.05 (*n* = 2 × 10).

### Field experiments

**Figure 3:**
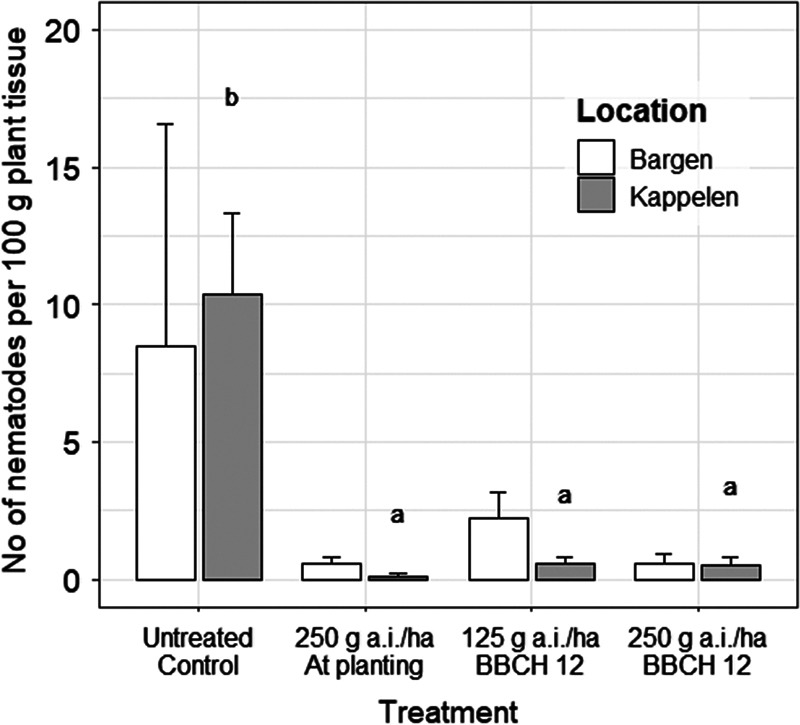
Effect of fluopyram applied in-furrow at planting at 250 g a.i./ha, or in-band at BBCH 12 (two leaves unfolded) at either 125 or 250 g a.i./ha in the 2016 field trials at Bargen and Kappelen on the number of *Ditylenchus dipsaci* per plant 62 days post-planting. Different letters over bars indicate significant differences at *α* = 0.05 according to the Tukey multiple comparisons of means (*n* = 4). Field locations were not compared to each other.

**Figure 4:**
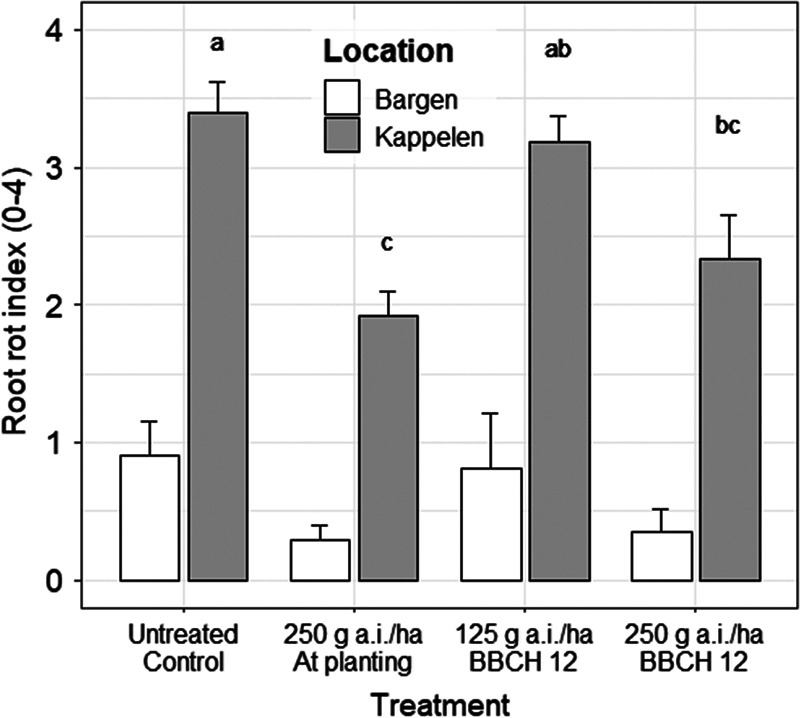
Effect of fluopyram applied in-furrow at planting at 250 g a.i./ha, or in-band at BBCH 12 (two leaves unfolded) at either 125 or 250 g a.i./ha in the 2016 field trials at Bargen and Kappelen on the rotting severity of the sugar beet root. Rotting index: 0 = no rot visible; 1 = ≤ 10% of rotten root; 2 = 11 to 33% of rotten root; 3 = 34 to 66% of rotten root; 4 = ≥ 67% of rotten root based on total root volume. Different letters over bars within a location indicate significant differences at *α* = 0.05 according to the Tukey multiple comparisons of means (*n* = 4). Field locations were not compared to each other.

At 27 dpp, fluopyram applied at planting at 250 g a.i./ha showed a significant reduction in the number of *D. dipsaci* per seedling compared to the non-treated control at the Kappelen site (Table 3). Similar numbers of *D. dipsaci* per plant were found in all untreated plots (untreated control and plots designated for post-planting application at BBCH 12), indicating a homogenous infestation throughout the field. At 62 dpp, all fluopyram applications in Kappelen resulted in a significantly lower number of *D. dipsaci*/100 g plant (Fig. 3). At the second site, in Bargen, sugar beet plants from untreated plots tended to show higher numbers of *D. dipsaci* per plant at 62 dpp compared to fluopyram treated plants, but differences were not significant (Fig. 3). At harvest at both sites, fluopyram was not effective in reducing the final population densities of *D. dipsaci* in the soil and in the plant at any rate or application time. All treatments showed a *Pf*/*Pi* > 2 of *D. dipsaci* in soil. The root-rot index, determined at harvest, was high in Kappelen with 3.4 for untreated plots. Only the high application rate of fluopyram (250 g a.i./ha), either at planting or post-planting (BBCH 12), reduced the root-rot index significantly (Fig. 4). In Kappelen, crown rot symptoms caused by *Rhizoctonia solani* (AG-2IIIB) were observed in all four treatments, with a trend to higher symptoms expressions on untreated plants.

**Table 3.** Effect of fluopyram applied in-furrow at planting at 250 g a.i./ha, or in-band at BBCH 12 (two leaves unfolded) at either 125 or 250 g a.i./ha in the 2016 field trials at Bargen and Kappelen on the number of *Ditylenchus dipsaci* (± SE) per plant 27 days post-planting and 1 day before the post-planting application (BBCH 12) compared to untreated plants.

Treatment/site	Bargen	Kappelen
Untreated control	4.4 ± 3.7	28.8 ± 11.3
125 g a.i./ha BBCH 12	4.6 ± 2.1	22.0 ± 6.8
250 g a.i./ha BBCH 12	4.4 ± 3.5	29.0 ± 9.9
250 g a.i./ha at planting	1.0 ± 0.2	1.8 ± 1.7^a^**
	ns	

**Notes:** ns, non-significant. Field locations were not compared to each other. ^a^** indicates a difference from the other treatments within the same field, according to the Tukey multiple comparisons of means at *α* = 0.05 (*n* = 4).

## Discussion

This study demonstrated the limited potential of fluopyram to control *D. dipsaci* population development and its related damage to sugar beet. Fluopyram did affect the motility of *D. dipsaci*, which depended on the concentration of the active ingredient and the time of exposure. However, the 24 h EC_50_ value was more than 20 times higher for *D. dipsaci* in our study compared to *M. incognita* and *R. reniformis* (Faske and Hurd, 2015). It is not uncommon for *D. dipsaci* to show higher tolerance to nematicide/nematistatic compounds compared to other nematode species. *Ditylenchus dipsaci* motility was more tolerant to fluensulfone compared to *Pratylenchus penetrans, P. thornei*, and *Aphelenchoides fragariae* (Oka, 2014). Recovery in the motility of *D. dipsaci* was evaluated after 72 h because the majority of nematodes were still motile at 24 h for most of the fluopyram concentrations. There was no increase in motility in *D. dipsaci* after exposure to fluopyram for 72 h followed by rinsing, suggesting that the compound is nematicidal to this nematode. However, the absence of recovery of *D. dipsaci* may be explained by the long exposure time in contrast to the exposure time used for other nematode species, which partly recovered when removed from fluopyram after shorter exposures. Faske and Hurd (2015) reported nematode recovery in motility greater than 50% for *M. incognita* and *R. reniformis* 24 h after nematodes were rinsed and removed from a 1 h fluopyram exposure. Nematode recovery of motility was between 12.5 and 275% 24 h after *M. incognita* were rinsed and removed from a 24-hr fluopyram exposure (Wram and Zasada, 2019). Schleker et al. (2019) reported complete recovery of *Heterodera schachtii* J2, even at high a.i. concentrations. Therefore, caution is required when interpreting the nematicidal effect of fluopyram on *D. dipsaci* because of the high EC_50_ values and prolonged exposure to the active ingredient in this study. Oka and Saroya (2019) reported irreversible immobilization of *M. javanica* and *M. incognita* after 48 hr exposure to 4.0 µg/ml fluopyram, whereas 17 hr exposure caused reversible immobilization. The first observations after exposure to fluopyram for 24 h suggest a low nematicidal effect on *D. dipsaci* in our experiment, whereas previously reported experiments suggested a nematistatic effect of fluopyram after a short exposure time. Shorter exposure to fluopyram would have been more appropriate to investigate recovery in the motility of *D. dipsaci* in our experiment. The exposure time is a decisive factor to investigate nematistatic or nematicidal activity of fluopyram (Oka and Saroya, 2019).

The high 24-hr EC_50_ value may explain the lack of efficacy of fluopyram at reducing *D. dipsaci* penetration on sugar beet seedlings in controlled conditions. The infectivity experiment investigated doses up to 10 µg/ml fluopyram, whereas the 24-hr EC_50_ value on *D. dipsaci* was 46.7 µg/ml. The rapid motility of *D. dipsaci* and the capacity to invade seedlings in few hours likely limited the effect of fluopyram to control *D. dipsaci* penetration into sugar beet seedlings. Furthermore, nematode inoculation through the first pair of true leaves of the seedlings reduced contact of fluopyram with the nematodes when the compound was applied to soil at planting (Dahlin et al., 2019; Ji et al., 2019). The development of an assay system for *D. dipsaci*, including a soil nematode inoculation method, will help to understand better the effect of fluopyram on infective nematodes at planting. We, as well as others (Kühnhold et al., 2006), were not successful at inoculating *D. dipsaci* into the soil. The field experiments showed the efficacy of fluopyram in reducing *D. dipsaci* penetration on sugar beet seedlings 27 days after soil application of the compound. The rainy spring in 2016 favored an optimal migration of *D. dipsaci* toward the plants and diffusion of fluopyram into the soil. The low temperature in the field slowed nematode penetration into the plant and, thus, increased exposure time to fluopyram. The at- and post-planting applications in the field showed a similar number of *D. dipsaci* per plant. Both application times at the 250 g a.i./ha rate showed significantly fewer nematodes per plant than the untreated control in Kappelen 62 dpp. The potential systemic activity (not documented for sugar beet) of fluopyram in sugar beet may have allowed a.i. accumulation in underground plant tissues (Matadha et al., 2019) leading to an interruption of nematode activity in post-plant treated seedlings.

In glasshouse and field experiments, fluopyram did not affect the reproduction of *D. dipsaci* in the plant at harvest. The low long-term efficacy of fluopyram for controlling nematode populations has been shown with *M. incognita* and *H. schachtii* (Dahlin et al., 2019; Hajihassani et al., 2019; Schleker et al., 2019). Based on the field trials, fluopyram appears to be effective in suppressing populations of *D. dipsaci* in the first weeks after the application. At 0.5 mg a.i./kg soil, corresponding to 376 g a.i./ha, a half-life for fluopyram of 64.2 days suggests a limited duration of nematicidal activity (Zhang et al., 2014). At 0.3 mg a.i./kg soil, Dong and Hu (2014) reported a half-life for fluopyram of 15.8 to 34.8 days and 6.48 to 6.60 days in soil and watermelon, respectively. At 0.2 and 0.1 mg a.i./kg soil, a half-life for fluopyram in onion were 8.85 and 9.12 days, respectively (Patel et al., 2016). The rainy spring of 2016 may have also decreased the half-life of the active ingredient in the two fields. Breakdown of nematicides occurs most rapidly in moist soils (Haydock et al., 2013). Fluopyram application at sowing may have killed *D. dipsaci* before infection or disrupted chemoreception and the ability of the nematode to find sugar beet seedlings (Haydock et al., 2013). The post-plant application treatment appeared to have caused nematodes to leave sugar beet because there were fewer nematodes found at 62 dpp than at 27 dpp (Schomaker and Been, 2013). The high reproduction capacity of *D. dipsaci* (Yuksel, 1960) resulted in a rapid increase of the nematode in the treated plots leading to similar populations to the untreated plots. The high populations of the nematode in the soil at harvest are partially due to the exodus of the nematode from heavily infected plant tissue and the absence of food (Schomaker and Been, 2013).

Fluopyram applied post-planting (BBCH 12) partially controlled the development of secondary infections following a *D. dipsaci* attack. In Kappelen, the high application rate of fluopyram (250 g a.i./ha) significantly reduced the root-rot index compared to the untreated control. However, the root-rot index observed in the plots applied with a high rate of fluopyram still led to 11 to 33% yield losses, rendering the sugar beet production unprofitable (Jenni, 2016, personal communication). Crown-rot symptoms caused by *Rhizoctonia solani* (AG-2IIIB), observed in Kappelen, were visible on the four treatments. The wounds caused by *D. dipsaci* favor the infection of *R. solani* (AG-2IIIB) on sugar beet (Hillnhütter et al., 2011). The fungicide activity spectrum of fluopyram, including several pathogens belonging to Ascomycota, may not be sufficient to control the development of basidiomycete pathogens such as *Rhizoctonia* spp. (Veloukas and Karaoglanidis, 2012). In Bargen, the low pathogen pressure from *D. dipsaci* explained the absence of statistical difference in root-rot between the untreated and treated plots.

Fluopyram has shown effective control on root-knot nematodes (*Meloidogyne* spp.) on different crops. However, *D. dipsaci* showed lower susceptibility to fluopyram, as demonstrated by the high EC_50_ in motility tests. Fluopyram was effective in the field at preventing *D. dipsaci* penetration into sugar beet seedlings. However, the short-term efficacy of the active ingredient only delayed the development of the nematode population and the occurrence of root-rot. In fields presenting a low *D. dipsaci* and secondary pathogens pressure, fluopyram may be an effective short-term control strategy. The lack of efficacy of fluopyram in reducing *D. dipsaci* populations in the soil does not indicate a sustainable role of the active ingredient for managing this nematode. Combining the use of fluopyram with cultural methods, such as crop rotation and late sowing, may allow profitable sugar beet production in infected areas in the short-term until other more effective management options are available, such as resistant cultivars.

## References

[ref001] Avenot, H. F., Thomas, A., Gitaitis, R. D., Langston, D. B., Jr. and Stevenson, K. L. 2012. Molecular characterization of boscalid- and penthiopyrad-resistant isolates of *Didymella bryoniae* and assessment of their sensitivity to fluopyram. Pest Management Science 68:645–651. 22076736 10.1002/ps.2311

[ref002] Beeman, A. Q. and Tylka, G. L. 2018. Assessing the effects of ILeVO and VOTiVO seed treatments on reproduction, hatching, motility, and root penetration of the soybean cyst nematode, *Heterodera glycines*. Plant Disease 102:107–113. 30673448 10.1094/PDIS-04-17-0585-RE

[ref003] Dahlin, P., Eder, R., Consoli, E., Krauss, J. and Kiewnick, S. 2019. Integrated control of *Meloidogyne incognita* in tomatoes using fluopyram and *Purpureocillium lilacinu*m strain 251. Crop Protection 124:104874.

[ref004] Dong, B. and Hu, J. 2014. Dissipation and residue determination of fluopyram and tebuconazole residues in watermelon and soil by GC-MS. International Journal of Environmental Analytical Chemistry 94:493–505.

[ref005] Duncan, L. W. and Moens, M. 2013. “Migratory endoparasitic nematodes”, In Perry, R. N. and Moens, M. (Eds), Plant nematology 2nd ed, Ghent: CABI, 144–178.

[ref006] Dutta, T. K., Powers, S. J., Gaur, H. S., Birkett, M. and Curtis, R. H. C. 2012. Effect of small lipophilic molecules in tomato and rice root exudates on the behaviour of *Meloidogyne incognita* and *M. graminicol*a. Nematology 14:309–320.

[ref007] European and Mediterranean Plant Protection Organization. 2013. PM 7/119 (1) nematode extraction. EPPO Bulletin 43:471–495.

[ref008] European Food Safety Authority. 2013. Conclusion on the peer review of the pesticide risk assessment of the active substance fluopyram. EFSA Journal 11:3052.

[ref009] Faske, T. R. and Hurd, K. 2015. Sensitivity of *Meloidogyne incognita* and *Rotylenchulus reniformis* to fluopyram. Journal of Nematology 47:316–321. 26941460 PMC4755706

[ref010] Fielding, M. J. 1951. “Observations on the length of dormancy in certain plant infecting nematodes”, In Rheinard, E. G. (Ed.), Proceedings of the Helminthological Society of Washington. Vol. 2, The Helminthological Society of Washington, Washington, DC, 110–112.

[ref011] FOAG. 2019. Dénomination commerciale: Moon Privilege: Index des produits phytosanitaires. Federal Office for Agriculture, available at: https://www.psm.admin.ch/fr/produkte/6828 (accessed April 23, 2019).

[ref012] Hajihassani, A., Davis, R. F. and Timper, P. 2019. Evaluation of selected nonfumigant nematicides on increasing inoculation densities of *Meloidogyne incognita* on cucumber. Plant Disease 103:3161–3165. 31545697 10.1094/PDIS-04-19-0836-RE

[ref013] Haydock, P. P. J., Woods, S. R., Grove, I. G. and Hare, M. C. 2013. “Chemical control of nematodes”, In Perry, R. N. and Moens, M. (Eds), Plant nematology 2nd ed., CABI, Ghent, 459–480.

[ref014] Hillnhütter, C., Albersmeier, A., Berdugo, C. A. and Sikora, R. A. 2011. Synergistic damage by interactions between *Ditylenchus dipsaci* and *Rhizoctonia solani* (AG 2–2IIIB) on sugar beet. Journal of Plant Diseases and Protection 118:127–133.

[ref015] Hurd, K., Faske, T. R. and Emerson, M. 2015. Evaluation of Poncho/VOTiVO and ILeVO for control of root-knot nematode on soybean in Arkansas, 2014. Plant Disease Management Reports 9, N017 pp.

[ref016] Hutchinson, C. M., McGiffen, M., Becker, J. O., Sims, J., Hutchinson, C. and Ohr, H. 1999. Efficacy of methyl iodide soil fumigation for control of *Meloidogyne incognita*, *Tylenchulus semipenetrans* and *Heterodera schachtii*. Nematology 1:407–414.

[ref017] Ji, X., Li, J., Dong, B., Zhang, H., Zhang, S. and Qiao, K. 2019. Evaluation of fluopyram for southern root-knot nematode management in tomato production in China. Crop Protection 122:84–89.

[ref018] Jones, J. T., Haegeman, A., Danchin, E. G. J., Gaur, H. S., Helder, J., Jones, M. G. K., Kikuchi, T., Manzanilla-López, R., Palomares-Rius, J. E., Wesemael, W. M. L. and Perry, R. N. 2013. Top 10 plant-parasitic nematodes in molecular plant pathology. Molecular Plant Pathology 14:946–961. 23809086 10.1111/mpp.12057 PMC6638764

[ref019] Kiewnick, S. and Sikora, R. A. 2006. Biological control of the root-knot nematode *Meloidogyne incognita* by *Paecilomyces lilacinus* strain 251. Biological Control 38:179–187.

[ref020] Kim, J., Mwamula, A. O., Kabir, F., Shin, J. H., Choi, Y. H., Lee, J. -K. and Lee, D. 2016. Efficacy of different nematicidal compounds on hatching and mortality of *Heterodera schachtii* infective juveniles. The Korean Journal of Pesticide Science 20:293–299.

[ref021] Kühnhold, V., Kiewnick, S. and Sikora, R. A. 2006. Development of an in vivo bioassay to identify sugar beet resistance to the stem nematode *Ditylenchus dipsaci*. Nematology 8:641–645.

[ref022] Lenth, R. V. 2016. Least-squares means: The R Package lsmeans. Journal of Statistical Software 69:1-33.

[ref023] Matadha, N. Y., Mohapatra, S., Siddamallaiah, L., Udupi, V. R., Gadigeppa, S. and Raja, D. P. 2019. Uptake and distribution of fluopyram and tebuconazole residues in tomato and bell pepper plant tissues. Environmental Science and Pollution Research International 26:6077–6086. 30613891 10.1007/s11356-018-04071-4

[ref024] Mohotti, K. M., Prematunga, A. K., Herath, U. B., Amarasena and P. G. D. S. 2018. “New prophylactic nematicide for integrated nematode management in tea”, New prophylactic nematicide for integrated nematode management in tea. Talawakelle: The Tea Research Institute of Sri Lanka, pp. 2–11.

[ref025] Oka, Y. 2014. Nematicidal activity of fluensulfone against some migratory nematodes under laboratory conditions. Pest Management Science 70:1850–1858. 24452922 10.1002/ps.3730

[ref026] Oka, Y. and Saroya, Y. 2019. Effect of fluensulfone and fluopyram on the mobility and infection of second-stage juveniles of *Meloidogyne incognita* and *M. javanica*. Pest Management Science 75:2095–2106. 30843368 10.1002/ps.5399

[ref027] Oka, Y., Shuker, S. and Tkachi, N. 2012. Systemic nematicidal activity of fluensulfone against the root-knot nematode *Meloidogyne incognit*a on pepper. Pest Management Science 68:268–275. 21842527 10.1002/ps.2256

[ref028] Patel, B. V., Chawla, S., Gor, H., Upadhyay, P., Parmar, K. D., Patel, A. R. and Shah, P. G. 2016. Residue decline and risk assessment of fluopyram + tebuconazole (400SC) in/on onion (*Allium cepa*). Environmental Science and Pollution Research International 23:20871–20881. 27484201 10.1007/s11356-016-7331-8

[ref029] Ritz, C. and Streibig, J. C. 2005. Bioassay analysis using R. Journal of Statistical Software 12:1–22.

[ref030] Ritz, C., Baty, F., Streibig, J. C. and Gerhard, D. 2015. Dose-response analysis using R. PloS One 10:e0146021. 26717316 10.1371/journal.pone.0146021 PMC4696819

[ref031] Schleker, S., Rist, M., Matera, C., Damijonaitis, A., Collienne, U., Matsuoka, K., Twelker, K., Scharwey, M., Schlee, U. and Grundler, F. M. W. 2019. “Nematicidal or nematistatic! Mode of action of fluopyram in plant parasitic nematodes”, In Daub, M. (Ed.), Kurzfassungen der DPG-Arbeitskreis-Tagung Nematologie. Vol. 47. Elsdorf: Deutsche Phytomedizinische Gesellschaft e.V. pp. 3–4.

[ref032] Schomaker, C. H. and Been, T. H. 2013. “Plant growth and population dynamics”, In Perry, R. N. and Moens, M. (Eds), Plant nematology 2nd ed, Belgium: CABI, 301–330.

[ref033] Silva, J. D. O., Loffredo, A., da Rocha, M. R. and Becker, J. O. 2019. Efficacy of new nematicides for managing *Meloidogyne incognita* in tomato crop. Journal of Phytopathology 167:295–298.

[ref034] Veloukas, T. and Karaoglanidis, G. S. 2012. Biological activity of the succinate dehydrogenase inhibitor fluopyram against *Botrytis cinerea* and fungal baseline sensitivity. Pest Management Science 68:858–864. 22262495 10.1002/ps.3241

[ref035] Wram, C. L. and Zasada, I. A. 2019. Short-term effects of sublethal doses of nematicides on *Meloidogyne incognita*. Phytopathology 109:1605–1613. 31033406 10.1094/PHYTO-11-18-0420-R

[ref036] Yuksel, H. S. 1960. Observations on the life cycle of *Ditylenchus Dipsaci* on onion seedlings. Nematologica 5:289–296.

[ref037] Zhang, Y., Xu, J., Dong, F., Liu, X., Wu, X. and Zheng, Y. 2014. Response of microbial community to a new fungicide fluopyram in the silty-loam agricultural soil. Ecotoxicology and Environmental Safety 108:273–280. 25105487 10.1016/j.ecoenv.2014.07.018

